# Sleeping under Insecticide-treated Nets to Prevent Malaria in Nigeria: What Do We Know?

**DOI:** 10.3329/jhpn.v31i2.16389

**Published:** 2013-06

**Authors:** Nkechi G. Onyeneho

**Affiliations:** Department of Sociology/Anthropology, University of Nigeria, Nsukka, Nigeria

**Keywords:** Insecticide-treated bednets, Malaria, Mosquito nets, Utilization, Nigeria

## Abstract

Malaria remains a public-health concern in Nigeria despite huge global investments in the production and distribution of insecticide-treated bednets (ITNs) to protect people from *Plasmodium falciparum* parasite. Information on the use of ITNs is needed for designing strategies for its effective use. Focus group discussions (FGDs) were conducted in communities from 3 geopolitical zones of Nigeria. The people had poor knowledge of malaria and mosquito bites, which resulted in wrong perception and misuse of the nets as door and window blinds to “protect entire household” since only two nets were given per household. The use of community structures (traditional leaders/village heads, youths, churches, and mosques) was suggested to ensure effective distribution of nets, sensitize, and monitor net-use in the communities. Health education would dispel misconceptions that ITNs could kill, curtail human fertility, and that local gin (*Kai-Kai*) would induce sleep and make one oblivious of mosquito nuisance.

## INTRODUCTION

Malaria currently accounts for nearly 110 million clinically-diagnosed cases per year, 60% of outpatient visits, and 30% of hospitalizations in Nigeria alone (1,**2**). Pregnant women and children are most at risk of malaria transmission and its effects (**3**). About 300,000 children die of malaria and over 30 million pregnancies threatened throughout Africa each year (2). It accounts for 11% of maternal mortality and 12-30% of mortality in children below 5 years in Nigeria, the hardest-hit country in Africa (4). Malaria in pregnant (MIP) women is a major risk factor of child death in the first month of life (5,6). It causes about 15% of maternal anaemia and about 35% of preventable low birthweight, which is a leading cause of neonatal mortality (7). MIP prevalence in Nigeria is estimated at 48% in 2000 and 2001 (8), and the prevalence in the first, second and third trimester is 37.5%, 47.3%, and 47.5% respectively (9).

The burden of malaria, its prevention and control remains a challenge despite the existence of effective technologies (10). Garner *et al.* (11) estimated that effective prevention of malaria with chloroquine prophylaxis or intermittent presumptive treatment (IPT) reduces the risk of low birthweight by as much as 43%. Fegan, *et al*. (12) also reported 44% reduction in mortality due to the use of insecticide-treated bednets (ITNs). In realization of the effectiveness of ITN against malaria, there have been improvements in the production of ITNs. Between 2008 and 2010, a cumulative total of 289 million ITNs were delivered to sub-Saharan Africa, enough to cover 76% of the 765 million persons at risk (13).

However, the use of ITN in Nigeria falls short of global targets (14). The 2008 Nigeria Demographic and Health Survey (NDHS) results indicate that 17% of households in Nigeria own a mosquito-net (treated or untreated), and 8% of households own more than one mosquito-net. Sixteen percent of households own at least one ever-treated mosquito-net, and 7% own more than one ever-treated mosquito-net. The average number of ITNs per household was less than one, which could be attributed to a weak supply and distribution mechanism (1).

Net distribution in recent years has been epileptic, with only a few Local Government Areas (LGAs) targeted in various states. This has made it impossible to attain full saturation in any one area. The approach since 2009 has been to start afresh a coordinated strategy to deliver 2 nets to every household across the country through a series of stand-alone campaigns to achieve universal coverage. In 2010, the World Bank Booster-supported states (Kano, Jigawa, Bauchi, Gombe, Anambra, Akwa Ibom, and Rivers) conducted net campaigns, and health workers distributed free nets to households. The aim was to promote net-use in households, especially among pregnant women and children below five years of age.

However, involvement of communities in malaria control raises several complex questions about the perceptions of malaria, its causes, prevention, and control. Answers to these complex questions reside in the people who are the end-users and the heartbeat of all efforts to control malaria through promotion of ITN-use. Often, the views of the people are missing from the so-called “people-centred” discussions on malaria control intervention. This paper documents outcomes of the study to assess people's knowledge of malaria and their perceptions of ITN in preventing malaria.

## MATERIALS AND METHODS

### Study design and sites

The study was designed to allow a description and analysis of community perceptions/views of ITN distribution and malaria control in Nigeria, employing qualitative inquiry in Anambra, Bauchi, and Rivers states

### Study population and sampling

The study population consisted of residents of communities where ITN distribution took place. Persons aged 18 years and above, who are statutorily adults in Nigeria, were included in the study. The number of FGDs conducted in each state is contained in Table 1. The FGDs were conducted during the dry season.

In total, 3 LGAs and 18 communities were randomly selected from each state. The FGDs were staggered across each state to cover 3 groups of adults: females, males, and youths respectively. This gave a total of **27** FGDs in the entire study. Table 2 presents the senatorial zones, LGAs, and communities visited in each state. Each FGD session consisted of an average of 9 participants (ranging from 8 to 10). The convenience sampling technique was employed in the selection of participants.

**Table 1.** Focus group discussions on malaria control by locality

Group	LGA 1		LGA 2		LGA 3		Total
	Urban	Rural	Urban	Rural	Urban	Rural	
Adult males (30+ years)	1		1			1	3
Adult females (30+ years)		1	1		1		3
Youths (18-29 years)		1		1	1		3
Total	1	2	2	1	2	1	9
Total FGD sessions in the 3 states=27							

LGA=Local government area

Before commencement of the study, the state malaria control officer took the researcher to the community leaders and introduced the mission. The community leaders, in turn, mobilized the community members and informed them that the officials from the state capital have come to discuss issues relating to their health in the community. They were also informed that some of them will be needed for focus group discussions with the researcher.

### Instrument and method of data collection

The instrument for data collection was an FGD guide. It explored such themes as people's perceptions of malaria, its causes, prevention, and treatment as well as the people's access to and use of ITNs. The discussions were tape-recorded and photographed, where permission was granted.

The discussion sessions were informal. The team spent approximately one week in a state for activities which included mobilization of the groups, actual discussion, and data-editing. The FGD facilitators were from the respective states sharing the cultural background with the participants.

### Method of data analysis

Analysis of the data placed emphasis on the interpretation, description, and recording/writing of what was actually said. In going through the transcripts, phrases with contextual or special connotations were noted and pulled out as illustrative quotes in developing the ethnographic summaries. To do this, relevant themes were developed for the coding and sorting of the qualitative data, and Atlas.ti (version 5.0) software was used in managing the qualitative data.

**Table 2.** Communities, LGAs, and senatorial zones visited in each of the study states by locality

State	Senatorial zone	LGA	Community	Locality
Anambra	Anambra Central	Awka South	Ukwu Orji	Urban
			Nibo	Rural
	Anambra North	Oyi	Oyi	Urban
			Nteje	Rural
	Anambra South	Nnewi North	Okpuno Egbo	Urban
			Umudim	Rural
Bauchi	Bauchi Central	Ganjuwa	Ganjuwa	Urban
			Miya	Rural
	Bauchi North	Katargum	Azare	Urban
			Bidir	Rural
	Bauchi South	Bauchi	Kofan Madaki	Urban
			Dumi	Rural
Rivers	Rivers East	Ikwere	Isiokpo	Urban
			Elele	Rural
	Rivers West	Bonny	Bonny Island	Urban
			Light House	Rural
Rivers Southeast	Khana	Bori	Urban
			Bua Khani	Rural

### Ethics

The procedures followed in the study were in accordance with the ethical standards of the research ethics committee on human experimentation of the University of Nigeria and received ethical clearance from the University of Nigeria Teaching Hospital Ethical Review Board.

The community leaders gave their consent for the study to be conducted in their respective communities. The participants were briefed on the procedures of the discussion, and their consent was received before the discussions commenced.

## RESULTS

### Common health problems

The discussions opened with a review of the common health problems in the communities. Several health problems were mentioned, albeit with some variations across states and even within and among LGAs in the states. Malaria was featured very prominently, irrespective of state, LGA, or the community. Samples of quotes to illustrate the findings are as follows:

We have something like rashes on the body, onchocerciasis, malaria, and eye problem, which are too much in our community. [Participant: FGD, Male adult; Oyi LGA, Anambra]

Malaria is more because it can come in different ways until that person goes for test and they confirm it to be malaria while that person must have been taking treatment for another sickness. So, there are different malarias. [Participant, FGD, Female adult, Urban Anambra]

However, the youths in Oyi LGA believed that malaria had a downward trend due to government commitment to its eradication:

In our community, malaria is common but from my own view, malaria is reducing gradually because the Government is making effort to eradicate it. [Participant: FGD, Youth, Urban Anambra].

As was the perception in Anambra, people in Bauchi and Rivers believed that malaria had a downward trend:

Before, malaria was so much but now the level has gone down. [Participant: FGD, Female adult, Rural Bauchi]

#### Knowledge of malaria in the communities

All participants were well-aware of malaria as a major health problem. This is reflected in the misconceptions about malaria captured in some quotes. In relation to other health problems, malaria was described as a ‘big’ sickness because it has the tendency of manifesting with other health problems and attacks may occur several times in one month. Following are some quotes that illustrate the magnitude of malaria as a problem in the communities:

Malaria is a very ‘big’ sickness in this community. Most children are brought to the hospital; this is because of malaria. If there are five children on admission in the hospital, three of them are suffering from malaria. [Participant: FGD, Youth, Rural Bauchi]

Knowledge of the cause of malaria was, however, mixed and appeared generally low. While some of the FGD participants knew that the malarial parasite is transmitted through mosquito bites, others mentioned factors, like eating too much oily food, working too hard, and exposure to sun, dirty environments, water-bearing plants, like plantain, coco-yam, and water-leaf, around the home.

Some even believed that the problem with mosquito was limited to the troublesome bite and disturbance in sleeping. Thus, once they are able to sleep, the mosquito bites will have no major source of concern. For instance, a male participant in an FGD session in rural Rivers state had this to say:

I don't think malaria is a problem because once you drink enough *Kai-kai* (local gin), you will sleep and will not know if mosquito is biting you. [Participant: FGD, Male adult, Rural Rivers]

Other participants in different FGD sessions, however, noted that malaria incidence could be attributed to multiple factors:

Malaria is associated with many factors. It is not caused by a single factor. The environment in which people are living can cause malaria. Stagnant water and the kind of water we are drinking all cause malaria. [Participant: FGD, Male adult, Rural Rivers]

A few, however, knew that mosquitoes transmit malarial parasite when they bite people. For example, a female participant in an FGD session in urban Rivers noted, “What causes malaria is when you are bitten by mosquito.” Another female participant in an FGD session in the urban area said, “What I know is that it is *Anopheles* mosquito, the female mosquito that bites. When it is infected and it bites another person that person will be sick.”

In Bauchi, like the situation in Rivers and Anambra states, the prevalence of malaria is attributed to environmental factors. A participant in a female FGD session in the rural area stated:

The main cause is stagnant water and bad drainage everywhere, poor hygiene and filthy environment. Formally, the government people fumigate and spray the gutters with chemicals that kill mosquitoes. But now they have stopped, and the mosquitoes breed in these gutters and stagnant water and so spread malaria in the community…In this community, we do a lot of farm work, and working under this harsh sun causes malaria, and we are not keeping our environment clean. [Participant: FGD, Youth, Rural Anambra]

Environmental factors, like stagnant water, bushes, and dirt around the homes do have some bearing on the transmission of malaria because of the suitable breeding sites they provide for mosquitoes, the vector of *Plasmodium* parasite. It is important to note that, within the youth groups in three states, mosquito bite was mostly mentioned as the major cause of malaria and its transmission:

What causes malaria is nothing but mosquito and too much work. In our community, old women do a lot of farm work which increases the rate of malaria because the mosquito bites them in the bush. [Participant: FGD, Youth, Rural Anambra]

Knowledge of the cause of malaria appeared higher among the youths. In Rivers state, however, the youths stressed other environmental factors, like stagnant water, water-bearing plants, exposure to sunlight, activities of oil companies, like gas flaring, oil exploration, and bad drinking-water as the major causes of malaria, as their older counterparts did:

One of the causes of malaria is our water. We do not have borehole or any kind of pipe-borne water; so, it is affecting the community with high fever and any other kind of fever, you know. [Participant: Male adult, Rural Rivers]

This may be attributed to the peculiar nature of the environment in Rivers state, with much of gas flaring and oil spillage, among other oil-related activities in the state. All the participants recognized that there is no age segregation in susceptibility to mosquito bites and malaria attacks. According to a youth in Bori, Rivers state, “mosquito does not know age and bites everybody.” In Anambra, the youth in Nnewi North stressed that “mosquito will not ask for your age before biting you.”

Pregnant women and children below five years were recognized as the group most affected by malaria. This is true for all the groups and study sites. According to a participant in an FGD session with adult females in Anambra state, “the blood of the pregnant women is being shared with the foetus while the under-five child's blood is not yet strong.”

There were some participants who did not believe in any differences in the impact of malaria on any group. According to a male FGD participant in Rivers, “malaria does not know age.”

### Management of malaria

Generally, there is evidence of prompt management but not necessarily appropriate treatment of malaria at the onset of signs and symptoms. Participants' first action is to visit the nearest chemist to ‘mix’ drugs. Only when this fails, participants need to visit healthcare centre or hospital. Although a few mentioned going for laboratory tests, this depended largely on the financial buoyancy of the concerned person or the carers. A male FGD participant in rural Rivers state said:

In our community, we do not have a healthcare centre. So, anytime we have fever, we just go to the chemist and ask for drugs but we do not even know what is exactly wrong because we do not go for tests…. We rush to any nearby chemist to purchase all these drugs. We do not know the one that is adulterated and the one that is real. As villagers, we just take them like that. [Participant: FGD, Youth, Rural Rivers]

**Figure.**
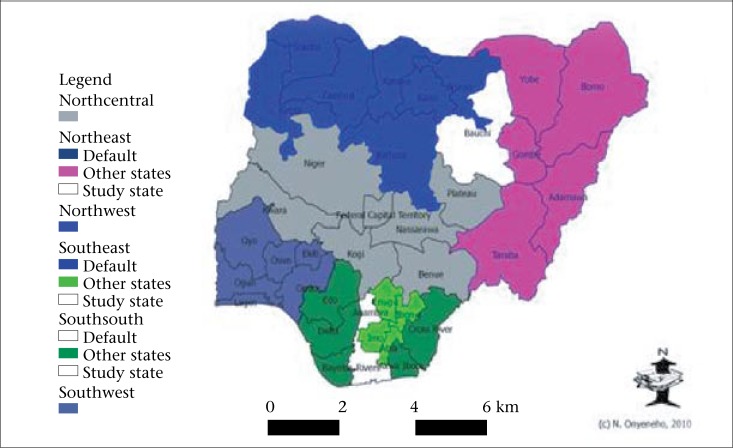
Map of Nigeria: States included in bednet campaign in white

Traditional medication was popular among participants in Anambra state. They argued that current antimalarial drugs in the market were no longer effective. Some participants in the male adult group in Anambra state would only use traditional medication for the treatment of malaria. A participant said:

In this community, anybody who has malaria handles this in a native way; we do what our ancestors taught. Now the person will go to hospital. But there are native herbs, and you can get some leaves and cook these and cover yourself with wrapper or mat, and the heat from those cooked leaves (local steam bath) will cause you to sweat very well. After that, you take bath with this water, drink the water, and it cures malaria very well. [Participant: FGD, Male adult, Rural Anambra]

Similar opinions were expressed by women in Bauchi and Rivers state as well as by men in Nnewi North, who felt that the native medicines were natural and unadulterated.

However, some dissenting views were expressed by those who did not believe in the use of traditional medication because it has no standard dosage. A female adult in an FGD session in Rural Anambra argued that the sick person goes to hospital for treatment.

### Prevention of malaria

Malaria is seen by all participants as a very serious ailment. According to participants, malaria is so serious that “it can make somebody go mad”; “It kills a lot of people”; “It makes people to be very weak.” For them, “nobody sees malaria as cough or catarrh because malaria is very serious.” Other typical quotes include:

Malaria is a very serious sickness and is very common. It is one of the sicknesses that disturb our people. It is a part of the household properties we have, due to its commonness in the community, although unfortunately it is a sickness. It is very common. [Participant: FGD, Male adult, Urban Anambra]

The participants believed that dirty environment is a heaven for mosquitoes. So, keeping the environment clean should be a top priority in the effort to protect people from malarial attack. Apart from environmental cleanliness, some participants do little or nothing because there is very little one can do to protect oneself since one cannot carry a mosquito-net around. Participants in Anambra felt that a net could protect one inside the room but not in the farms and other places. In the words of a rural youth of Anambra in an FGD session, “We prevent malaria by using that net but we cannot carry the net to the farm or market. Other solutions need to be found.”

In Rivers state, participants held the view that ITN is good but some expressed dissatisfaction with the method of its distribution, especially as the youths are excluded in the distribution. According to a participant in the youth group, “My father uses one of the ITNs, and my mother uses the other”; so, children and the youths do not have protection from malaria. In an FGD session with rural youths in Anambra, it was advised that the nets should be given to every household, and these should be given to young boys and girls as well as those who have a place to sleep. This was supported by other participants in FGD session.

Participants in all the states agreed that the net works well for malaria prevention but also insisted that the nets were grossly inadequate for the number of people requiring those. Mainly participants from Anambra state mentioned the use of mosquito-coil and shelltox for protection from malaria; those living in the rural settings were used to burning certain leaves, the smoke of which has the ability to drive away mosquitoes, for protecting themselves from malaria. In Rivers, however, there were participants who took drugs to protect themselves from malaria. A female participant in an FGD session stated:

What I know is coartem, and you should clear the bush around your house and not allow stagnant water remain around your house because if there is stagnant water, mosquitoes will breed there and will enter the house and bite. And if you have net, you should get your family to sleep inside.

### Sources of ITNs and costs

In Anambra state, all participants got their net through the healthcare centres nearest to them while those in Rivers state got those from either the healthcare centre or the house of the village head, where there were no healthcare centres, or primary school, or church close to the people. Interestingly, none of the participants claimed to have paid any money before getting the ITNs. Following are some illustrative quotes on the sources and cost of ITN in the different communities visited:

We got it (ITN) from Government through our Mai Angwa (community head). [Participant: FGD, Female adult, Urban Bauchi]

We got net from the hospitals or designated distribution points. [Participant: FGD, Female adult, Rural Bauchi]

I got net the time they distributed in Nteje healthcare centre and they gave two to a family. [Participant: FGD, Male adult, Rural Anambra]

We got the net through the healthcare centre in Bonny. [Participant: FGD, Male adult, Urban Rivers]

### Perceptions of ITNs

In all three states visited, the ITNs were viewed as very good by all participants. The nets were accepted by the people as those were capable of preventing malaria. Majority of participants had nets and used them accordingly. However, the nets were not easily available or accessible. Some participants who did not get the net when it was distributed were not able to access the nets. There is a general agreement among the participants that, although the nets are good, the supply was not enough. All participants agreed that the nets were “free gifts from the Government; so, we all got those free of charge without paying even one Naira (~US$ 0.01).”

Desirability for the net was obvious but this was marred by wrong perceptions that emanated from inadequate sensitization. Although a good number of participants (who had the nets) claimed to use those, there are others who did not use those for various reasons. For instance, there were mothers who reserved the net for their unborn grandchild-ren whom they expected from their current teenage daughters. There were also those who cut up the nets and used those as door and window blinds because the two nets given to each family were not adequate for the needs of very large families. So, in order to protect everyone, the nets were fixed onto the windows and doors. Some did not use the nets because of wrong perceptions. In Anambra and Rivers state, there were people who did not use the net and said, “I heard that one man slept inside the net and vomited blood” and “I heard that it causes skin irritation because the chemical is too much.”

In the same vein, there are people in Bauchi state, who thought that the nets were laced with “birth control chemicals.” Generally, the nets were still in very high demand. Following are some quotes that support the people's appreciation and demand for more nets, irrespective of the study site and group:

Truly, this ITN was distributed in this community. There are some people who did not get the ITN. This ITN has been of immense benefit to us. Before, mosquito disturbed us so much but now it does not disturb us so much again. We put one ITN inside the room. We put one in the veranda (balcony). When we enter the room without children, we are inside one. If we come out to stay in the veranda we are inside the ITN. So, we are now saying bye-bye to mosquitoes. [Participant: FGD, Female adult, Urban Bauchi]

People who want it are many. There are people who will like to sleep inside it, with heat so that mosquitoes do not bite him/her. It depends on the person's body. [Participant: FGD, Male adult, Urban Anambra]

### Use of ITNs in the communities

The success of any programme depends not only on the efficacy of the intervention provided under ideal and controlled conditions but also on achieving optimal use by the target population. In the opinion of participants, those who got the nets were using these because they wanted to protect themselves from malaria. A youth in an FGD session in a rural community in Rivers state said, “I believe people are using it.” In another FGD session with urban females in Rivers state, a participant said, “Yes, some people sleep under nets, some do not because they complain of heat.” This was only one of the reasons for the low usage of the nets. During the cold periods, people were quite happy to use the nets because these produced heat. Countering the belief that the net induced heat, a participant in an FGD session with female adults in Khana LGA, Rivers state, said she enjoyed the warmth from the nets, particularly during the rainy season. In her words:

You know that you can never please the world. There is nothing you can do that people will praise you…. As rain falls, if you sleep under this net you will enjoy sleep. I like this net very much … and I sleep very well inside it, and I will like Government to bring more.

Reasons for the low usage also included inadequacy of the nets for the number of people in the home, ignorance regarding instruction for use, fear of the unknown as well as spousal disagreement. In some households, men monopolize the use of the nets while the women and children sleep without the nets. Following are sample quotes that illustrate these points:

We use the ITN to cover the windows with nails and everywhere, through which mosquito can enter the room [Participant: FGD, Female adult, Rural Anambra]

Some people are using it but some are not because, in some families, they are ten in number, and there are only two nets; so, only two people will use.

Wrong usage of the net was also reported. Some of those who used the net wrongly lived with the illusion that they used it often. One way they used it wrongly was to fix it to the doors and windows as a way of making every member of the household benefit from the net. A participant in an FGD session with youths in rural Anambra said:

Some people use it for the real purpose of preventing mosquito bites. In short, I will say people wish to use it to protect themselves against mosquito bites but the problem is the wrong usage. Some simply use it as window and door blinds still with the purpose of warding off mosquitoes but this is the wrong approach.

Typically, participants in some of the FGD sessions, who received the nets but fixed them on their windows and doors, argued that they used their nets everyday.

### Participants' opinion on distribution of ITNs in the communities

In Anambra state, participants were satisfied with the way the distribution was done but to ensure wider coverage, participants were of the opinion that there should be an announcement so as to make people stay home to collect their net-cards. This is because most people who did not get the net were not at home to collect net-cards when these were distributed. Participants were particularly pleased with innovation of tearing the nets out of the packets and giving the bare nets to the people to encourage them to use it instead of storing or selling them. In urban communities of Rivers state, the distribution was also applauded but, in the rural communities, participants who were predominantly farmers suggested that the distribution should be done on Sundays through their various churches. Similar views were also expressed in Bauchi:

Before they gave us ITN, they first collected our names and the number of wives we have…. They then gave us cards. On the day of the distribution, we gave them the cards, and the ITN was given to us for free at the hospital. If you do not have card, you are not given ITN. [Participant: FGD, Male adult, Rural Bauchi]

### Suggestions on ways of improving distribution of ITNs in the communities

While participants in Bauchi appreciated the current distribution method, they also suggested that using the heads of the communities would ensure wider coverage because the community heads knew each person in the community and would ensure that no one would collect more than he/she was entitled. In Rivers state, the churches were believed to be better points for distribution because farmers, fishermen, and women are found at home on Sundays. Participants in Anambra are in support of using community leaders:

What can be done so that the net can reach everybody is that there are leaders in all streets, and these nets should be given to them to share with their people. If they bring these to the healthcare centre, not everybody will know about it [Participant: FGD, Male adult, Urban Anambra]

### Suggestions on ways of making people use ITN in the communities

Health education is suggested to be the remedy to making people use ITN. A participant in Khana, an adult male, captured this clearly when he said, “many people have net but they are not using it.” The quote below reflects other concerns:

It is true that people complain of heat but giving them health education either through church, school or market. will make them see that enduring heat is better than having malaria. Then, the net should be shared through the community leaders. There should also be community sanitation to remove stagnant water which attracts mosquito. [Participant: FGD, Male adult, Rural Rivers]

## DISCUSSION

Malaria is a major health problem in the three states investigated. The magnitude of malaria is reflected in the sentiments, through which the people expressed their concerns about malaria. To prevent, treat, and/or manage malaria, the people resort to all sorts or practices, like taking local herbs and roots while a few use orthodox medication. This is akin to the argument of the health belief model (HBM) (15). HBM argues that the perceived severity of any problem as well as the perceived effectiveness of available solutions influence the adoption of intervention.

People are beginning to appreciate the effectiveness of insecticide-treated nets in the prevention of mosquito bites and malaria transmission. Some even attest to the fact that, with the nets, malaria has now a downward trend. They endeavour to collect their free nets for this purpose. Those who succeeded in collecting the nets which were given free of charge under the World Bank Malaria Booster programme, put those into usage. Some, in appreciation of the efficacy of the nets, reserved those for their grandchildren yet unborn, others used those as door and window blinds in the hope that all in the household would be protected since only two nets were given to a household that may have twelve or more persons.

Some even discouraged the use of net and recommended reliance on *Kai-kai* (local gin) instead. For this category of persons *Kai-kai* will put one to sleep and make the person oblivious of the biting menace of mosquito, implying that the only problem with mosquito is the disturbing ‘ringtone’.

These rumours and misconceptions about the nets could be attributed to poor health education and sensitization on the nets. The health workers are often in a hurry to share the nets without taking time to educate the people on appropriate net-usage. The people are left to speculate on the nets. These findings largely corroborate results from similar studies conducted in other African countries (16-18)

The involvement of community leadership structure and local organizations, like churches, community heads, and the youths, was strongly advocated to promote acceptance and compliance. The community resource persons can also educate the people on proper use, monitor, and encourage ITNs.

### Conclusions

Communities should be involved in all the processes of prevention that are built on net-use. Access to nets alone does not translate into effective net-use. Community members are better positioned to dispel misconceptions about ITNs, assist in net installation, and monitor usage. This is in line with the community-directed intervention (CDI) strategy, widely acknowledged as successful in the delivery of health interventions in African countries (19-22).

## ACKNOWLEDGEMENTS

This study received financial and logistic support from the World Bank Group in Nigeria. Supports received from the state focal persons for malaria in the three states covered are equally acknowledged. Community leaders who gave consent for the study in the respective communities are appreciated.
